# Exploring microbial signatures in sarcoidosis: etiological and therapeutic implications of host–microbiota interactions

**DOI:** 10.3389/fimmu.2026.1735877

**Published:** 2026-01-28

**Authors:** Luigi Rizzi, Giovanni Larizza, Patrizia Suppressa

**Affiliations:** 1 Internal Medicine Unit “Miulli” Hospital, Acquaviva delle fonti, Italy; 2 Head of Internal Medicine Unit “Miulli” Hospital, Department of Medicine & Surgery, LUM University Giuseppe Degennaro, Casamassima, Italy

**Keywords:** gut microbiota, interstitial lung diseases, lung microbiota, rare diseases, sarcoidosis

## Abstract

Sarcoidosis is a chronic inflammatory disease that can potentially affect any organ. From a pathogenetic standpoint, it is characterized by the formation of chronic granulomatous inflammation, which defines its histological hallmark. Unlike its pathogenetic mechanisms, the etiology of sarcoidosis remains poorly understood. Environmental triggers—such as viruses, bacteria, fungi, or exposure to damp environments—have been hypothesized as risk factors in genetically predisposed individuals. Recently, increasing attention has been given to the role of the gut microbiota in the development of various metabolic and autoimmune disorders. At this regard, the primary aim of this review has been to critically explore the potential role of the gut and lung microbiota in the onset of sarcoidosis. We examined current and available evidence regarding the composition of the pulmonary and intestinal microbiota in sarcoidosis, identifying possible differences compared to other interstitial lung diseases and trying to find potential correlations with the etiological and prognostic features of the disease. Furthermore, potential therapeutic implications for the treatment of sarcoidosis have been assessed, with a focus on the possibility of modulating the microbiota of affected individuals as a novel approach.

## 1 Introduction

Sarcoidosis is a rare inflammatory disease characterized by the formation of non-necrotizing granulomas in the organs involved. It can potentially affect any organ, being lungs the most common involved site. The course of the disease is variable, ranging from acute self-limiting forms to chronic ones. The incidence ranges from 1 to 11–24 or 18–71 cases per 100.000 individuals per year, depending on geographical area (Asian countries, Scandinavian countries and African Americans, respectively), while the average age of onset is 40–55 years (1). One of the lesser-known aspects of sarcoidosis is its etiology: microorganisms, environmental factors and inorganic materials have been considered as potential triggers for inflammation in genetically predisposed individuals (1); on the other hand, in the context of an immune system dysregulation, a possible role of autoimmunity has also been hypothesized (2). Recently, several studies focused on the role that microbiota may play as both a therapeutic target and a contributor to the pathogenesis of various pulmonary diseases; the term “microbiota” refers to the whole ecological community of commensal, symbiotic, and pathogenic species, including bacteria, viruses and fungi that share the human bodily space, as well as the interaction between these microbes and the host (3). Based on this premise, the aim of this review is to analyze the role that the microbiota may play in the etiology of sarcoidosis, as well as its therapeutic implications.

## 2 Microbiota in sarcoidosis patients

### 2.1 The historical ‘traces’ of the association between sarcoidosis and microbial agents

Several studies historically support the hypothesis that certain microorganisms may play a role in the pathogenesis of sarcoidosis. As known, sarcoidosis and tuberculosis share several clinical and histopathological features; for this reason, one of the most studied microorganisms in relation to sarcoidosis is Mycobacterium tuberculosis: some meta-analysis suggested that Mycobacterium tuberculosis infection is significantly associated with sarcoidosis (4). Similarly, a correlation between Propionibacterium acnes and sarcoidosis was found: according to meta-analysis-derived evidence, infection with Propionibacterium acnes increases the risk of developing sarcoidosis by approximately 19-fold (4).

### 2.2 Lung microbiota in sarcoidosis

In healthy individuals, the lung microbiota typically comprises Prevotella, Streptococcus, Veillonella, Bacteroidetes, and Firmicutes species (3). According to several studies, these microorganisms play a crucial role in preserving and sustaining local homeostasis, as they interact with lung immune cells such as alveolar macrophages and dendritic cells, defending from chronic inflammation through the recruitment and activation of regulatory T-cells, M2-polarized macrophages, and tolerogenic dendritic cells (5).

In the context of sarcoidosis, there are several evidence demonstrating an altered lung microbiota. Clarke et al. analyzed the microbial environment of 93 sarcoidosis patients in different specimens (lymph node biopsies, broncho-alveolar lavage – BAL - and spleen): they found an enrichment of Cladosporiaceae fungi and bacterial lineages Corynebacterium and Rhodocyclaceae in lymph node tissues and an enrichment of Aspergillus fungi on BAL (6). It is noteworthy that Corynebacterium can cause granulomatous diseases such as granulomatous lobular mastitis. An enrichment of Aspergillus fungi on BAL has been detected also in patients with Löfgren’s syndrome, suggesting a potential role of Aspergillus in the pathogenesis of sarcoidosis (7).

According to Zimmerman et al. results, the BAL of 71 sarcoidosis patients resulted enriched of Atopobium spp. and Fusobacterium spp (8), whereas an enrichment of Shewanella, Pseudomonas, Acinetobacter, Lactobacillus, Prochlorococcus and Propionibacterium acnes was found in lymph node biopsy specimens of 17 patients with sarcoidosis by Zhao et al. (9).

Another study by D’Argenio et al. identified in Bacteroidetes, Proteobacteria, Firmicutes, and Fusobacteria the most prevalent phyla in BAL samples of sarcoidosis patients, while Garzoni et al. highlighted the prevalent presence of Prevotellaceae, Acidaminococcaceae, and Streptococcaceae in their lower airway microbiota (10, 11).

Finally, further studies identified a prevalence of Prevotellaceae, Burkholderiaceae, Streptococcaceae, Enterobacteriaceae, Reyranellaceae and an abundance of taxa belonging to Corynebacterium and the Neisseria family (12).

### 2.3 Gut microbiota and sarcoidosis

Studies that clarify the relationship between gut microbiota and sarcoidosis are lacking. Lee et al. compared the composition of gut microbiota between 50 sarcoidosis patients and 50 controls. In the sarcoidosis patients, the analysis revealed an increase of 27 specifical species and a reduction of 239 species when compared to controls. Moreover, results showed that the particular microbial environment was related to general immune activation, monocyte activity, and heme-related inflammation (13).

## 3 Microbiota in other interstitial lung diseases

### 3.1 Idiopathic pulmonary fibrosis

Several studies highlighted that lung microbiota of idiopathic pulmonary fibrosis (IPF) patients significantly differs from healthy people. In particular, an enrichment of Haemophilus, Streptococcus, Neisseiria, and Veillonella genera was demonstrated; moreover, the presence of specific Streptococcus or Staphylococcus species above a certain threshold has been proven to be associated with a faster-progressing disease (14, 15). According to Zhu et al., not only the lung microbiota but also the intestinal microbiota may play a role in the pathogenesis of IPF: the results of a two-step, two-sample Mendelian randomization study showed that 12 particular taxa (Bacillales, Gastranaerophilales, Selenomonadales, Family XIII, Bacteroidaceae, Bacteroides, and Actinomyces, Bifidobacterium, Oscillibacter, Ruminococcus gnavus, Subdoligranulum, Veillonella) of gut microbiota and 8 circulating inflammatory proteins (CCL11, CXCL6, CXCL9, CCL8, CCL7, NRTN, STAMPB, and TGFa) may be causally related to the pathogenesis of IPF (16).

### 3.2 Systemic sclerosis

Systemic sclerosis is a multiorgan immune-mediated rheumatic diseases characterized by vasculopathy, fibrosis of the skin and internal organs. It may be responsible of an interstitial lung disease (ILD) that often progresses to fibrosis (14). In this clinical context, studies underlined the relationship between gut microbiota and ILD: according to Caimmi et al. results, among 129 outpatients with systemic sclerosis, those with ILD presented higher values of fecal calprotectin, suggesting that gut dysbiosis was a risk factor for ILD onset and progression (17).

### 3.3 Rheumatoid arthritis

Rheumatoid arthritis (RA) is a chronic, autoimmune inflammatory disease that can cause irreversible joint damage; pleuro-pulmonary manifestations of RA include interstitial pulmonary fibrosis, organizing pneumonia, lung nodules, bronchiolitis, bronchiectasis and pleural effusions. Even in this case, several studies suggested a relationship between an infectious trigger and the development of the disease. In particular, oral microbiota may contribute to the disease onset, as Prevotella and Leptotrichia species were typically found in patients with new-onset RA and absent in controls (18). Regarding lung microbiota, studies identified a BAL microbiota significantly less diverse and abundant than healthy persons, with a reduction of taxa such as Paraprevotellaceae, Chryseobacterium, Burkhordelia, Actinomycetaceae, and Spirochaetaceae (19).

### 3.4 Sjögren’s syndrome

Sjögren’s syndrome is an autoimmune disorder that affects salivary, conjunctival, and pharyngeal mucosal glands; pulmonary involvement is characterized by the development of an interstitial disease (16). Studies about changes in patients’ microbiota are contradictory: from one hand, there are evidence regarding a lower Firmicutes/Bacteroidetes ratio in gut microbiota of Sjögren’s syndrome patients; from the other hand, studies collectively indicated that significant differences in gut, oral or ocular microbiota in Sjögren’s syndrome were lacking, probably suggesting that the changes in the microbiota might be only a consequence of mucosa dryness (20). Moreover, studies regarding the composition of lung microbiota in this specifical disease are absent.

### 3.5 Systemic lupus erythematosus

Systemic lupus erythematosus is a multisystemic autoimmune disease that can affect skin, heart, kidney, musculoskeletal district, central nervous system and lungs. Among pleuropulmonary manifestations, systemic lupus erythematosus can lead to an interstitial lung disease, sometimes complicated by fibrosis (14). In this case, several studies highlighted the presence of gut dysbiosis, characterized by an abundance of Rhodococcus, Eggerthella, Klebsiella, Prevotella, Eubacterium, Flavonifractor, and Incertae sedis, and a reduction of the genera Dialister and Pseudobutyrivibrio (21). Moreover, there are evidence of a direct relationship between gut microbiota dysbiosis and the disease severity (22). Otherwise, studies about lung microbiota are lacking.

### 3.6 Dermatomyositis

Dermatomyositis (DM) is an idiopathic inflammatory myopathy characterized by the inflammation of skeletal muscle and skin, commonly complicated by interstitial lung disease (14). Lou et al. found a prevalence of eight genera including Corynebacterium, Aeromonas and Achromobacter in BAL of 14 DM-ILD, compared with 27 RA patients and 18 controls (23). Regarding gut microbiota, studies have demonstrated an increase of Bacteroidetes in DM patients and a significant prevalence of Proteobacteria and a reduction of Christensenellaceae and Ruminococcaceae in the ILD subgroup (24).

### 3.7 Silicosis and hypersensitivity pneumonitis

In 18 patients with silicosis, an ILD caused by inhalation of silica, Zhou et al. demonstrated a reduced bacterial diversity of gut microbiota and an expansion of Proteobacteria in comparison with 21 healthy subjects (25).

Regarding hypersensitivity pneumonitis, the hypothesis that gut microbiota may influence the clinical course of the disease arises from the evidence that some antibiotics able to modulate gut microbiota such as streptomycin can worsen the disease severity (14). Currently, no studies regarding the relationship between lung microbiota and hypersensitivity pneumonitis are available.

## 4 Critical synthesis of the current evidence

Regarding sarcoidosis, the studies analyzed aren’t able to trace a common microbial signature (**Table 1**). For example, convergent evidence about the abundance of Fusobacteria in the BAL of sarcoidosis patients (8, 10) are not completely reliable due to a different sample analyzing method. Moreover, the main study that highlights the evidence about the abundance of Aspergillus regards a small sample of patients affected by Löfgren syndrome, leading to non-generalizable results. Similarly, the evidence regarding the abundance of Propionibacterium acnes arise from the analysis of lymph nodes samples of 17 sarcoidosis patients (9): in this case, the different sample type doesn’t allow a comparison with other studies in which BAL is the most frequent sample type analyzed.

**Table 1:** Synthesis of the studies regarding microbiome in sarcoidosis and other ILD.

Disease	Author and year	Main findings	Sample size	Sample type	Method	Limitations
*Sarcoidosis*	Clarke et al. (2018) (6)	Cladosporiaceae, Corynebacterium, Rhodocyclaceae, Aspergillus, Cladosporium	n=93 sarcoidosis; n=72 non-sarcoidosis controls; n=150 environmental controls	Lymph nodes, BAL, Kveim and spleen tissues	Illumina HISEq. 16S rRNA V1V2 and ITS1F/1TS2 Shotgun sequencing WGS	Sampling not done at the onset of the disease; role of microbes enrichment not clear in sarcoidosis pathogenesis; low resolution analysis
	Greaves et al. (2021) (7)	Aspergillus nidulans	n=9 sarcoidosis with Löfgren syndrome; n=4 sarcoidosis without Löfgren syndrome; n=3 control subjects	BAL	TCR clustering using Grouping of Lymphocyte Interactions by Paratope Hotspots (GLIPH) algorithm	sample size; study limited to Löfgren syndrome
	Zimmerman et al. (2017) (8)	Atopobium spp. Fusobacterium spp.	n=71 sarcoidosis patients; n=10 healthy subjects; n=15 IPF patients	BAL	Roche G454 FLX titanium technology and chemistry sequencer; 16S rRNA V1V2; 16S rDNA	Lack of knowledge about previous usage of corticosteroids; low resolution analysis
	Zhao et al. (2017) (9)	Propionibacterium acnes Propionibacterium granulosum Shewanella Pseudomonas Acinetobacter Lactobacillus Prochlorococcus	n= 17 sarcoidosis; n=8 tuberculosis; n=11 controls	Lymph nodes	Illumina MiSeq. 16S rRNA V4	Sample size Sample type low resolution analysis
	D’Argenio et al. (2018) (10)	Bacteroidetes Firmicutes Fusobacteria Proteobacteria	n=10 sarcoidosis; n=9 other ILD	BAL	Roche 454 Sequencing 16S rRNA V4V6	Sample size low resolution analysis
	Garzoni et al. (2013) (11)	Prevotellaceae, Acidaminococcaceae, Streptococcaceae	n=5 idiopathic interstitial pneumonia (IIP); n=6 non-IIP; n=7 sarcoidosis; n=6 Pneumocystis pneumonia; n=9 healthy controls	BAL, oropharyngeal swab	Roche 454 Sequencing 16S rRNA	Sample size low resolution analysis
	Gupta et al. (2021) (12)	Prevotellaceae, Burkholderiaceae, Streptococcaceae, Enterobacteriaceae, Reyranellaceae, taxa belonging to Corynebacterium and the Neisseria family	n=13 exacerbated chronic obstructive pulmonary disease (ECOPD); n=14 stable COPD; n=8 ILD; n=8 sarcoidosis	BAL	16S rRNA	Sample size low resolution analysis
*Idiopathic pulmonary fibrosis*	Richter et al. (2009) (15)	Haemophilus, Streptococcus, Neisseiria, and Veillonella	n=33 patients with Wegener granulomatosis; n=22 IPF; n=8 normal controls	BAL	16S rRNA sequencing; 454 Pyrosequencing; Quantitative BALF culture	Sample size low resolution analysis
	Zhu et al. (2025) (16)	Bacillales, Gastranaerophilales, Selenomonadales, Family XIII, Bacteroidaceae, Bacteroides, and Actinomyces, Bifidobacterium, Oscillibacter, Ruminococcus gnavus, Subdoligranulum, Veillonella	n=14306	GWAS Catalog (genome-wide association studies) for gut microbiota	Two-sample, two-step mendelian randomization study.	Data source (public dataset)
*Rheumatoid arthritis*	Scher et al. (2012) (18)	Prevotella, Leptotrichia	n=31 new-onset rheumatoid arthritis; n=34 chronic rheumatoid arthritis; n=18 healthy controls	Oral samples	16S rRNA V1-V2; 454/pyrosequencing	Sample size low resolution analysis
	Scher et al. (2016) (19)	Reduction of Paraprevotellaceae, Chryseobacterium, and Burkhordelia, Actinomycetaceae, and Spirochaetaceae	n=20 rheumatoid arthritis; n=10 sarcoidosis; n=28 healthy controls	BAL	16S sequencing analysis	Sample size, low resolution analysis
*Systemic Lupus Erythematosus*	He et al. (2020) (21)	Abundance of Rhodococcus, Eggerthella, Klebsiella, Prevotella, Eubacterium, Flavonifractor, and Incertae sedis; reduction of the genera Dialister and Pseudobutyrivibrio	n=21 systemic lupus erythematosus; n=10 healthy volunteers	Fecal sample	16S gut microbiome sequencing	Sample size, low resolution analysis
*Dermatomyositis*	Lou et al. (2022) (23)	Corynebacterium, Aeromonas and Achromobacter	n=14 dermatomyositis with ILD; n=27 rheumatoid arthritis; n=18 controls	BAL	16S rRNA amplification and sequencing	Sample size, low resolution analysis
	Bae et al. (2022) (24)	Increase of Bacteroidetes and Proteobacteria; reduction of Christensenellaceae and Ruminococcaceae	n=36 dermatomyositis; n=26 healthy controls	Fecal sample	16S rRNA gene sequencing of the V4 region by Illumina MiSeq	Sample size, low resolution analysis

Moreover, a particular microbial signature can’t be intercepted even comparing sarcoidosis with the other ILD or within the ILD subgroup.

From a methodological point of view, the studies analyzed show several issues. In the first place, sample size is generally too small, while sample type often differs among studies; in the second place, also the analysis methods are different among studies and show several limitations: for example, the 16s rRNA sequencing method, one of the most frequently used analysis techniques, shows a low taxonomic resolution and inability to gain functional and metabolic information. Moreover, results may not always take into account some anamnestic factors that can potentially modulate microbial signature: the temporal distance between the onset of the disease and the enrollment in the study; the possible influence of some therapeutic strategies on microbiota; the presence of comorbidities. Furthermore, the evidence of a clear relationship between a specific microbial signature and the pathogenesis, the evolution or the prognosis of the disease is generally lacking: in other words, an overall evaluation of the studies included in the review does not allow to clarify if microbiota could be considered a causal factor rather than a consequence of the presence of the disease. Finally, the lacking of a longitudinal design of the studies affects the reliability of the results. For all these reasons, the existing data can’t allow to establish a clear causal link between microbiota and sarcoidosis.

## 5 The gut-lung axis: a potential sarcoid-granuloma modifying factor

As examined, a peculiar composition or alteration of gut microbiota or lung microbiota may play a role in several diseases. The ability of the microorganisms to influence the onset and clinical course of certain diseases could be rooted in the complex system of the gut-lung axis. This can be imagined as an intricate, continuous communication network system between the pulmonary microbiota and the intestinal microbiota. In fact, there are evidence that modifications of newborns’ diet influence the composition of their lung microbiota, while fecal transplantation in rats induces changes in lung microbiota (26).

The correlation between the two systems is bidirectional, as the pulmonary microbiota can also influence the intestinal one. For example, it is supposed that influenza infection triggers may lead to an increased proportion of Enterobacteriaceae and decreased abundances of Lactobacilli and Lactococci in the gut or that lipopolysaccharide instillation in the lungs of mice can cause gut microbiota disturbances (27, 28).

Each of the two biological ecosystems interacts with the local immune system. On the one hand, the intestinal microbiota communicates with the intestinal mucosal system through pro-inflammatory and regulatory signals; on the other hand, the pulmonary microbiota plays a crucial role in the immune homeostasis of the lungs. In fact, evidence indicates that exposure of the lungs to commensal bacteria induces the expression of immune-regulatory molecules, reducing pro-inflammatory and pro-allergic stimuli (26). The cross-talk between gut and lung microbiota results in a significant long-reach immune impact. It is allowed by mesenteric lymphatic system, through which microorganisms may translocate across the intestinal mucosal barrier, reaching the systemic circulation and leading to modulate the lung immune response. In particular, short-chain fatty acids produced by bacterial fermentation are able to act in the lungs as signaling molecules on resident antigen-presenting cells to attenuate the inflammatory and allergic responses. Moreover, gut segmented filamentous bacteria, commensal bacteria colonizing the ileum of most animals including humans, can modulate the immune system regulating CD4+ T-cell polarization into the Th17 pathway; the latter is implicated in the immunological response of fungal infection and in lung autoimmune diseases (26).

One of the essential elements of the gut-lung interaction is represented by bloodstream: it can be considered the access route for microorganisms to migrate from one compartment to another. It is for this reason that the integrity of gut epithelial barrier is crucial to avoid the translocation of microbial elements: clinical conditions such as sepsis, critical illness or chronic inflammations lead to an alteration of intestinal epithelial barrier in terms of increased permeability (“leaky gut” phenomenon), promoting microbial circulation. It is interesting to note that certain lung viral infections may exacerbate the leaky gut syndrome, leading to a worsening of the viral infection itself induced by lung colonization of intestinal microbes. At this regard, several evidence support the hypothesis that the pathogenesis of certain pulmonary diseases such as sepsis or acute respiratory distress syndrome (ARDS) is influenced by the presence of mainly gut-derived bacteria (29). Ultimately, bloodstream may be considered the main pillar of the gut-lung axis interaction based on immunomodulatory and metabolite-mediated pathways (30). As previously mentioned, mesenteric lymphatic system is the connection interface between intestinal barrier and bloodstream: after gut injury due to a local or systemic stress, such as a splanchnic hypoperfusion secondary to septic shock, mesenteric lymphatic system may play an active role in the gut-lung axis inflammation response by contributing to the transport of gut-derived cytotoxic or inflammatory agents to the lungs, leading to an organ damage mediated by toll-like receptors or nitric oxide signaling pathways (31).

In light of these considerations, the gut–lung axis represents an intricate system of bidirectional communication with the local intestinal and pulmonary immune systems, resulting in the modulation of the systemic immune response. It therefore appears plausible that alterations in this immune ecosystem may lead to the development of pulmonary diseases (32).

These considerations are relevant in the context of the sarcoid granuloma formation, which can be considered the result of a complex interaction between genetic factors, environmental exposures and an exaggerated T helper-1 and T-helper 17 immune response. The immunological steps in granuloma formation begin with the exposure to an environmental factor such as infectious triggers; this leads to innate immune activation of macrophages and dendritic cells; then, an upregulation of the mechanistic target of rapamycin complex 1 (mTORC1) pathway leads to the formation of epithelioid cells, namely activated macrophages resembling epithelial cells, while an upregulation of serum amyloid A and heath shock proteins causes an effector T cell response mediated by toll-like receptor (TLR) 2, polarizing the immune response through T helper 1, T helper 17 and T helper 17.1 cells, under inflammatory stimulus of interleukin-12 (IL-12), IL-18, IL-6 and transforming growth factor-β. The resolution or the progression of the granuloma depends on the efficacy of CD-4 T-cell response: if peptide antigens are presented by human leukocyte antigen (HLA)-DR3 molecules on dendritic cells or macrophages and recognized by a specific T cell receptor (TCR) of a CD4-positive T helper-1 cell, an efficient immune response is generated, the antigen is eliminated and the granuloma resolves. Conversely, if the antigen recognition is not efficient, due for example to an antigen presentation process mediated by other HLA molecules, T-cell are not able to resolve inflammation, sarcoid granulomas continue to grow, leading to a chronic disease (1). Not by chance, HLA-DR3 positivity can be found in Löfgren syndrome, which can be considered a self-limiting form of sarcoidosis. Regarding infectious trigger, Mycobacterium tuberculosis and Propionibacterium acnes have been historically related to sarcoidosis, as previously stated (4). Although a causative role of these pathogens in sarcoidosis has not been clarified, it is reasonable to hypothesize that microorganisms may act as a trigger in the etiology of the disease (1).

In this perspective, an alteration of the gut-lung axis could potentially lead to the development of lung diseases: an alteration of the local microbiota can lead to the formation of specific microbial antigens that can stimulate an abnormal immune response in genetically predisposed individuals, supporting the onset of “dysimmune” disease such as sarcoidosis or “autoimmune” disease such as rheumatoid arthritis or systemic lupus erythematous.

## 6 Discussion

One of the most significant challenges in sarcoidosis concerns its etiology. Indeed, although the pathophysiological mechanisms underlying the disease have been well elucidated, current evidence regarding its etiology allows for the formulation of hypotheses rather than definitive conclusions. It is reasonable to assert that the disease may result from an exaggerated immune response in genetically predisposed individuals toward certain antigens, such as specific microorganisms (1). In light of these considerations, the analysis of the potential role of the intestinal and pulmonary microbiota in the etiology of sarcoidosis appears highly relevant.

Historically, two pathogens have been associated with an increased risk of developing sarcoidosis. The first is represented by Propionibacterium acnes. Several studies have identified the presence of this microorganism in sarcoid tissues such as lymph nodes or in the form of anti-Propionibacterium acnes IgA and IgG antibodies in the BAL of affected subjects (3).

The other microorganism potentially implicated in sarcoidosis onset is represented by Mycobacterium tuberculosis, namely the causative agent of tuberculosis. Despite the similar radiological and histopathological features of sarcoidosis and tuberculosis, it is unclear how this bacterium might contribute to the onset of sarcoidosis. This is also in light of the fact that studies conducted in this regard have rarely identified the presence of Mycobacterium tuberculosis in sarcoid tissue (3).

The aim of our review has been to investigate the possible role of microbiota in sarcoidosis etiology and clinical course. One of the most important considerations emerged from our results regards a common limitation of the studies investigating the microbiota: although peculiar compositional features of the gut and lung microbiota have been identified in sarcoidosis, the pathogenic role of each phyla present is often lacking. In particular, according to our review, studies highlighted, as a whole, an enrichment of sarcoidosis’ patients BAL from different microorganisms, including Cladosporiaceae fungi, Atopobium spp. and Fusobacterium spp, Bacteroidetes, Proteobacteria, Firmicutes, and Fusobacteria, Prevotellaceae, Acidaminococcaceae, and Streptococcaceae. For each of this pathogen, the demonstration of a clear pathogenetic role in sarcoidosis is lacking. Moreover, at this regard, an important consideration is that the results of the studies did not seem to identify a consistent homogeneous composition of the lung microbiota in patients with sarcoidosis: in other words, each study identified a specific and peculiar composition of lung microbiota in the patients enrolled. Given the relatively small sample sizes of the examined studies, this condition may hamper research progress and contribute to further fragmentation of the findings.

Another noteworthy observation arises from comparing sarcoidosis with other interstitial lung diseases: the lung microbiota composition appears to vary across the different conditions studied; even in this case, evidence for a definitive pathogenic role of the identified microbial flora is lacking.

As previously explained, the studies included in this review present other several limitations, ranging from small sample size to the different sample type among studies, the analysis methods, or the absence of a longitudinal design of the studies. For all these reasons, it is not possible to intercept pathogenetic causality links in the results of the analyzed studies. From our perspective, the pathogenic role of a specific microbial environment in the development of pulmonary diseases such as sarcoidosis may be mediated by the gut–lung axis. It is plausible that a particular alteration of the intestinal or pulmonary microbiota could trigger a targeted impairment of the immune system, initially local and later systemic, ultimately leading to the development of overt interstitial lung diseases, possibly associated with autoimmune conditions (25). With particular reference to sarcoidosis, evidence support the hypothesis that an altered composition of gut or lung microbiota can act as a source of microbes able to release pathogen-associated molecular patterns (PAMPs); the latter can activate monocytes or eosinophils by the interaction with pathogen recognition receptors (PRRs) like TLRs. In case of sustained activation, this process may lead to the recruitment of macrophages and to the consequent formation of giant cells and epithelioid cells; the secretion of cytokines such as IL-6, IL-12, IL-18 and tumor necrosis factor-α (TNF- α) sustains the granuloma formation process and its persistence (33).

These pathogenetic hypothesis find support in recent advances regarding host-pathogen interactions in tuberculosis: according to Nasiri et al. (34), Mycobacterium tuberculosis is a pathogen able to release Mycobacterium-derived lipids like phthiocerol dimycocerosates (PDIMs) and phenolic glycolipids (PGLs); the latter can alter granuloma microenvironment by masking bacterial surface antigens and impairing the recruitment of protective immune cells. The interference with TLRr leads to a pro-inflammatory cytokines suppression with consequent impaired antigen presentation and reduced macrophage activation. Moreover, trehalose dimycolate (TDM) plays a role in granuloma maturation and supports bacterial survival by impairing antibacterial activity of immune cells. Finally, Micobacterium tuberculosis upregulates PPM1A, a phosphatase that inhibits apoptosis in infected macrophages, allowing pathogens to persist in a protected intracellular area, eluding immune response and clearance.

Focusing on sarcoidosis, it would be interesting to investigate how microbiota can affect the course of the disease. According to the complex pathophysiological mechanisms underlying granuloma formation, it would be reasonable to hypothesize that an alteration of lung or gut microbiota may lead to an impaired immune response. This process could be at the basis of two phenomena: from one hand, the inability to an effective clearance of the microbial antigens may lead to a chronic and more severe form of sarcoidosis; from the other hand, the impairment of immune system could play an indirect role in the spreading of microbial antigens, contributing potentially to a multiorgan disease. Finally, there could be a certain relationship between the amount of microbial clearance and pharmacological therapeutical response.

For these reasons, research in the field of microbiota could pave the way for precision medicine in sarcoidosis treatment: if future studies will demonstrate that certain pathogens can be considered the cause of a chronic disease or treatment non-responsive disease, a microbiomics-based phenotyping of sarcoidosis patients may suggest specifical therapeutical target to improve prognosis.

In light of these considerations, future research should aim to explore how the microbiota can be modulated to regulate the immune system and restore its proper function.

At this regard, there are evidence about a successful use of antibiotics and corticosteroids for direct modulation of respiratory microbiota in treatment of chronic lung diseases, such as bronchiectasis, chronic obstructive pulmonary diseases (COPD) and interstitial lung diseases such as organizing pneumonia (35, 36).

A modulation of gut-lung axis can be achieved also acting on gut microbiota: probiotics and fecal microbiota transplantation may represent useful strategies to restore dysbiosis, improve gut mucosal barrier and enhance immunity response. Even in this case, data supports the hypothesis that probiotics can reduce inflammation in certain inflammatory diseases such as rheumatoid arthritis (37). Moreover, gut microbiota can be modified by diet: it has been proven that higher pro-inflammatory dietary intake (animal-based diet; reduced fiber content) is associated with various respiratory infections, COPD or reduced lung function (38).

However, specific evidence regarding the therapeutic role of probiotics, fecal microbiota transplantation or antibiotics in sarcoidosis are currently lacking.

For these reasons, it would be useful to plan observational studies in order to understand if the course of the disease in sarcoidosis patients may be changed through innovative and integrative therapeutic approaches such as probiotics, antibiotics, dietary interventions, or fecal microbiota transplantation. Preliminary studies should clarify which specific targets within the intestinal or pulmonary microbiome should be addressed in order to properly modulate the gut–lung axis.

In conclusion, the etiology of sarcoidosis remains an open challenge for scientific research. Several studies suggested a possible role of the microbiota in the onset of the disease. For this reason, investigating the gut–lung axis may provide the basis for the development of innovative and personalized medical therapies within this specific clinical context.
